# How Does Being Overweight Moderate Associations between Diet and Blood Pressure in Male Adolescents?

**DOI:** 10.3390/nu13062054

**Published:** 2021-06-15

**Authors:** Jia Yap, Hwei Min Ng, Meredith C. Peddie, Elizabeth A. Fleming, Kirsten Webster, Tessa Scott, Jillian J. Haszard

**Affiliations:** 1 Department of Human Nutrition, University of Otago, Dunedin 9054, New Zealand; yapji273@student.otago.ac.nz (J.Y.); hwei.ng@postgrad.otago.ac.nz (H.M.N.); meredith.peddie@otago.ac.nz (M.C.P.); liz.fleming@otago.ac.nz (E.A.F.); kirsten.webster@otago.ac.nz (K.W.); tessa.scott@otago.ac.nz (T.S.); 2 Division of Sciences, University of Otago, Dunedin 9054, New Zealand

**Keywords:** blood pressure, hypertension, adolescents, nutrition, obesity, New Zealand

## Abstract

Diet is one of the strongest modifiable risk factors for hypertension. In this study, we described the associations between dietary factors and blood pressure; and explored how weight status moderated these associations in a sample of New Zealand male adolescents. We collected demographics information, anthropometric, blood pressure, and dietary data from 108 male adolescents (15–17 years old). Mixed effects and logistic regression models were used to estimate relationships between dietary variables, blood pressure, and hypertension. Moderation effects of overweight status on the relationship between hypertension and diet were explored through forest plots. One-third (36%) of the sample was classified as hypertensive. Fruit intake was related to significantly lower systolic (−2.4 mmHg, *p* = 0.005) and diastolic blood pressure (−3.9 mmHg, *p* = 0.001). Vegetable and milk intake was related to significantly lower diastolic blood pressure (−1.4 mmHg, *p* = 0.047) and (−2.2 mmHg, *p* = 0.003), respectively. In overweight participants, greater vegetable and milk, and lower meat intake appeared to reduce the odds of hypertension. Certain dietary factors may have more prominent effects on blood pressure depending on weight status.

## 1. Introduction

Non-communicable diseases, in particular cardiovascular diseases (CVD), are the primary cause of premature deaths in the world [1]. An independent risk factor for CVD is high blood pressure (BP), which contributed to 19.2% of total deaths worldwide in 2019 [2]. In New Zealand (NZ), about one in five adults (21.6%) had high BP in 2018/19 which had remained consistent across four survey years [3]. It is well-documented that the prevalence of high BP increases steeply with age, thus, it is not surprising that NZ youth aged 15–24 years had the lowest prevalence of high BP compared to all older ages [3].

As males are more likely to develop high BP than women, understanding modifiable risk factors for men is crucial [4]. Based on the New Zealand 2018/19 Health Survey, men were 1.3 times more prone to having high BP than women (24.1% vs. 19.2%) [3]. This difference was even more pronounced in the NZ youth population where more males (7.9%) had high BP compared to females (3.4%) [3]. Similar patterns were also observed in other populations around the world [5,6,7].

Lifestyle choices such as adherence to a healthy diet (low sodium and saturated fat intake but high fruits and vegetables intake) [6,8,9], and maintaining a healthy weight [9] at early ages lowers the risk of developing high BP in adulthood. Prospective studies have consistently demonstrated that high BP at a young age persists into later life [7] and lifestyle factors are harder to modify in adulthood [8]. Therefore, adolescence may present an opportune window to intervene, particularly in those who are vulnerable to developing high BP.

The key modifiable risk factor in this age group is diet [4,8] but it is not known which dietary factors are associated with the greatest risk of high BP in adolescents of differing weight status. Obesity is also an important risk factor for high BP with an increasing number of studies indicating this association holds true in adolescents [6,10,11]. With just under half of the NZ youth population aged 15–24 (45.4%) being overweight or obese [3], the risk of developing high BP in this age group is of concern. Elucidating how risk factors such as diet and body weight interact at this critical period of life can help to inform prevention strategies for early onset of hypertension, and the establishment of lifelong healthy habits.

Due to the cognisance of increasing risk for high BP among the young male population, this study first aimed to describe dietary, demographic, and body-weight risk factors associated with high BP among a sample of NZ male adolescents. Secondly, this study aimed to provide better understanding on how dietary factors are related to the risk of hypertension by overweight status, through an exploratory moderation analysis.

## 2. Materials and Methods

### 2.1. Study Design

The SuNDiAL (Survey of Nutrition, Dietary Assessment, and Lifestyle) project 2020 was a cross-sectional study conducted in high schools from five regions in NZ aiming to explore the nutritional status and lifestyle habits among male adolescents aged between 15 and 18 years. Inclusion criteria were self-identified male, 15 to 18 years of age, who could speak and understand English. The study was approved by the Human Research Ethics Committee at the University of Otago, reference number H20/004), and was registered under the Australian New Zealand Clinical Trials Registry, reference number ACTRN12620000185965.

### 2.2. Recruitment

High schools across NZ with more than 400 male students were invited to participate via email. The SuNDiAL research teams visited participating schools and advertised the study to students through presentations and printed information. Interested students provided an email address and were sent a link to complete online consent and enrolment. Additional parental consent was required from students aged under 16 years. Recruitment was initially scheduled in two phases; February–April and July–September 2020; however, this was halted on the 23 of March 2020 due to coronavirus (COVID-19) lockdown.

### 2.3. Data Collection

Data were collected in three ways: a self-administered online questionnaire; an in-school visit; and a follow-up phone- or video-call. The online questionnaire, administered through Research Electronic Data Capture (REDCap) (Vanderbilt University), included demographic and health questions, as well as questionnaires about dietary habits, attitudes, and motivations for food choice. The in-school visit included a 24-h diet recall, anthropometric assessment, and BP measurement. The follow-up phone- or video-call was undertaken to collect a second 24-h diet recall.

### 2.4. Demographics 

Upon completing online consent, participants provided demographic information. This included home address and ethnicity. Socioeconomic status was measured based on New Zealand Deprivation Index 2018 deciles according to their home address, with 1 indicating the least deprived areas and 10 being the most deprived areas [12]. This was further categorized into low deprivation (score 1–3), moderate deprivation (score 4–7) and high deprivation (score 8–10). Self-identified ethnicity was assessed using the 2006 NZ census ethnicity questions and were prioritised into four ethnic groups based on the Ministry of Health’s priority classification system: Māori, followed by Pacific, then Asian, and finally New Zealand European and others [13].

### 2.5. Dietary Habits and Intake

Dietary habits were assessed with the Dietary Habits Questionnaire [14]. This asked, on average, how often the participant consumed various food groups (fruit, vegetables, milk, meat, plant protein, sweet drinks, and snacks), with 10 response options ranging from ‘never’ to ‘more than three times a day’. Some food groups were combined from smaller categories: ‘Meat’ was a combination of questions on red meat, pork, poultry, fish, seafood, and processed meat; ‘Plant protein’ was generated from questions about legumes, tofu, and vegetarian meat alternatives consumption; and ‘Snacks’ was a combination of questions about sweets and chocolate, sweet snacks (like biscuits and muesli bars), and savoury snacks (like crisps and crackers). Ordinal responses were first converted to average ‘times per day’ before combining foods together.

Dietary intake was assessed using two 24-h diet recalls performed on two non-consecutive days (preferably one weekday and one weekend day) a week apart. The first recall involved a physical interview at one of the school visits (or via phone/video call during COVID-19 lockdown for one school, *n* = 24 participants). The second recall was conducted via phone/video call. These were carried out according to a four-pass method as described elsewhere [15]. In brief, the participant and interviewer generated a quick list of all food and drinks consumed the previous day, followed by a detailed description of each food and beverage including brand, amount, and time consumed. Lastly, the interviewer and participant checked through the generated list together. Supermarket webpages were used to assist participants with reporting brands. Household measures and a photo booklet were used to help participants estimate portion sizes. Dietary intakes were entered into FoodWorks software (FOODfiles 2016-Version 01) for nutrient analysis and the multiple source method was applied to adjust for intra-individual variability [16]. Energy, fat, protein, carbohydrate, and fibre intake were adjusted to represent usual daily intakes. As energy intake varied considerably, fat, protein, and carbohydrate intake were converted to percent of energy intake using Atwater factors.

### 2.6. Anthropometry

Anthropometric measures were taken by trained SuNDiAL research teams using standard protocols [17]. Height and weight were collected in duplicate and a third measurement was taken if there was a 0.5 difference between the two initial measurements. Participants were required to remove shoes and heavy clothing before any measurements were carried out. Standing height was measured to the nearest 0.1 cm using a portable stadiometer (Seca 213 (Seca, Hamburg, Germany) or Wedderburn (Wedderburn, Sydney, Australia) stadiometer). Weight was recorded using the one of Medisana PS420 (Medisana, Nuess, Germany); Salter 9037 BK3R (Salter, Kent, United Kingdom); Seca Alpha 770 (Seca, Hambury, Germany); or Soehnle Style Sense Comfort 400 (Soehnle, Backnang, Germany) scales and recorded to the nearest 0.1 kg. Body Mass Index (BMI) was calculated as weight (kg) divided by height squared (m^2^). The BMI-for-age *z*-score was calculated with World Health Organisation growth reference data and was used to interpret weight status [18]. A BMI *z*-score of >+1 to ≤+2 was classified as overweight and >+2 as obese [18].

### 2.7. Blood Pressure

BP was measured using a digital blood pressure monitor (OMRON HEM-907; Omron Healthcare, Kyoto, Japan). Prior to having their measurements taken, participants were required to be in a seated position for 15 min, using an appropriately sized cuff with the artery indicator at the correct anatomical position. The BP monitor was set to take three measurements at one-minute intervals, and the average was recorded. The American Academy of Pediatrics cut off values were used to define elevated BP (≥120 mmHg systolic and <80 mmHg diastolic), and hypertension (>130/80 mmHg) [19].

### 2.8. Statistical Analysis

All statistical analysis was carried out using Stata 16.1 (StataCorp, College Station, TX, USA). As some participants with BP measurements did not complete a diet recall and/or the dietary habits questionnaire, demographic, and anthropometric differences between those with and without this data were assessed with t-tests for continuous variables (age and BMI *z*-score) and Fisher’s exact tests for categorical variables (deprivation, ethnicity, and weight status).

Mixed effects regression models were used to estimate the relationship between dietary variables and systolic and diastolic BP. BP was the dependent variable, dietary intake was the independent variable, and school was included as a random effect. Adjusted models were also generated that adjusted for age, ethnicity, and BMI *z*-score. Residuals of the models were plotted and visually assessed for heteroskedasticity and normality. The odds of hypertension associated with the demographic and dietary variables were estimated using logistic regression models with a sandwich estimator for school clusters. Adjusted estimates were also calculated controlling for age and BMI *z*-score.

To assess the moderating effect of overweight status on the relationships between diet and hypertension, forest plots were generated to illustrate the odds ratio (OR) and 95% confidence interval (CI) by whether participants were classified as overweight or not. Overweight participants were those who were classified as either overweight or obese. Testing for moderating effects involves the inclusion of an interaction term in the model, between the moderating variable and the independent variable. However, our sample size was too small to have the power to detect ‘statistically significant’ interactions for meaningful moderations (thus inflating the possibility of Type II error). Therefore, statistical tests of interaction were not carried out. Instead, the forest plots illustrate the possibility of clinically important moderation by examining if the odds ratios were markedly different between groups. If the estimates for each group are not dissimilar, then meaningful moderation is unlikely, regardless of sample size [20,21].

## 3. Results

Six high schools took part in the SuNDiAL 2020 study. A total of 146 students consented to the study but only 108 students attended an in-school visit and had their BP taken. Among these, 104 (96%) completed health and demographic questionnaire, 97 (90%) completed dietary habits questionnaire, and 88 (81%) completed at least one 24-h diet recall. Those who completed the dietary habits questionnaire (*n* = 97) were not noticeably different from the whole sample (*n* = 108) in terms of the demographics reported in Table 1 (all *p* > 0.1, analysis not shown). Similarly, in those who completed diet recalls (*n* = 88) there was no evidence of demographic differences from the whole sample (all *p* > 0.1, analysis not shown).

Demographics, weight status and BP are described in Table 1. Mean systolic BP (SD) was 124 mmHg (12.0) while diastolic BP (SD) was 65 mmHg (9.0). Just over half of participants were classified as having high BP (*n* = 63, 58.3%), while over one-third (*n* = 40, 37.9%) were classified as hypertensive.

Table 2 shows the mean difference and 95% CI in systolic and diastolic BP for dietary habits and intake. After adjusting for age, ethnicity, and BMI *z*-score, each serve of fruit per day was associated with 2.4 mmHg lower systolic (95% CI −4.0, −0.7) and 2.5 mmHg lower diastolic BP (95% CI −3.9, −1.1). Similarly, vegetable and milk were associated with a 1.4 mmHg (95% CI −1.8, −0.4) and 2.2 mmHg (95% CI −3.6, −0.8) lower diastolic BP, respectively.

Demographic and dietary predictors of hypertension are presented in Table 3. The odds of hypertension increased by 3.16 (95% CI 2.39, 4.19) for each additional year of age. Overweight individuals were 9.4 times (95% CI 2.89, 30.69) more likely to have hypertension than normal weight individuals. There were no significant associations between dietary intakes or habits and adjusted odds of hypertension.

**Figure 1:**
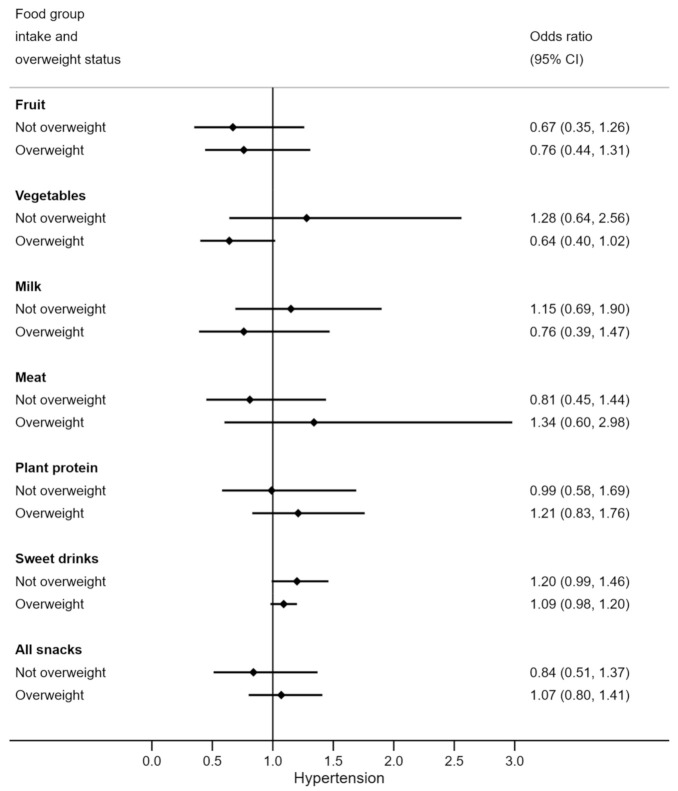
Odds of hypertension for each serve per day of various food groups, by overweight status. *N* = 97 (*n* = 33 overweight). Odds ratios are adjusted for age.

**Figure 2:**
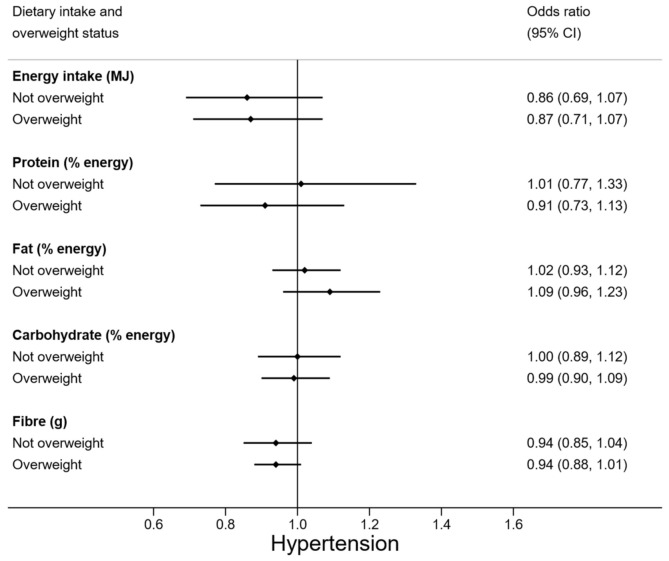
Odds of hypertension for energy, macronutrient, and fibre intake, by overweight status. *N* = 88 (*n* = 30 overweight). Odds ratios are adjusted for age.

Weight status appeared to moderate some relationships between diet and hypertension (Figure 1). Vegetable and milk consumption were more protective against the odds of hypertension in overweight adolescents (OR 0.64 (95% CI 0.40, 1.02) and OR 0.82 (95% CI 0.56, 1.19)) than in those who had normal weight. Meat consumption in overweight participants was non-significantly related to increased odds of hypertension by 1.34 times, but lower odds of hypertension among normal weight adolescents. Note that overweight adolescents had similar vegetable, milk, and meat intakes as their normal weight counterparts (Table A1 Appendix A). The relationship between macronutrient composition of the diet and hypertension did not appear to be moderated by weight status (Figure 2).

## 4. Discussion

High BP is a silent health problem that may go unnoticed in adolescents as they are not routinely measured. Among our sample of 15-to-17-year-old NZ male adolescents, more than half had high BP, with more than a third classified as hypertensive. Age and weight status were the strongest predictors of hypertension while fruit, vegetable, and milk intake, were shown to be associated with a lower BP. Exploratory analysis results indicated that weight status may moderate associations between diet and hypertension. Among overweight individuals, more servings of vegetables and milk, and a lower number of servings of meat a day appeared to be associated with decreased odds of hypertension, but not in normal weight adolescents.

Our results extend previous observations on the hypotensive effects of fruit and vegetable intake in adolescents and adults [22,23,24,25]. The exact mechanism of how this food group lowers BP is unclear; however, their high potassium and antioxidant contents are suggested to exert cardio protective effects via vasodilation [9,26]. Apart from this, they are also rich in fibre which has consistently demonstrated BP-lowering properties in prior studies [26,27,28,29]. The benefits of fibre intake on BP were somewhat supported by the current results. Likewise, the positive effects of milk intake on BP have also been observed in earlier research [30,31]. Evidence suggests that phosphorus, calcium, and magnesium (all found in dairy products) can exhibit BP lowering properties through the maintenance of cellular structure and function, which regulates the vascular tone [31].

While not statistically significant, our findings also support a relationship between sweet drink intake and BP as reported by earlier literature [32,33,34]. Note that previous studies only investigated the effects of sugar sweetened beverages on BP whereas ours included artificially sweetened beverages (diet drinks). Interestingly, despite the absence of fructose that was suggested to induce hypertension in sugar sweetened beverages [34], hypertensive adolescents reported higher diet drink consumption compared to their normotensive counterparts (mean intake = 1.9 per week compared to 0.6 per week). This was also reported in another study where diet drink consumers had higher BP than sugar sweetened beverage consumers and non-consumers (3.3 mmHg and 5.4 mmHg higher respectively) [35]. This may be mediated by salt intake as soft drink consumers (including diet drinks) tend to have diets higher in salt [35]. Alternatively, given the cross-sectional nature of this study, it is possible that participants with high BP changed to diet drinks in attempt to make healthier choices. Our study did not measure salt consumption in these adolescents hence we were unable to investigate this, but it is noted the sugar sweetened beverage consumption was low in this sample (less than one per day on average).

Similarly, our study did not identify any meaningful relationship between plant protein and BP as opposed to evidence from the INTERMAP study [36] and the PREMIER trial [24]. This may be due to the different type and quality of plant protein consumed two decades ago [24,36]. Our study included ultra-processed plant-based alternatives that are relatively new to the market and which were not analysed in both prior studies. Hence, this makes direct comparisons between the studies difficult due to the dissimilar nutritional components from varying plant protein sources.

Being overweight is a well-established independent risk factor for the development of hypertension [9,10,37,38,39] and this is consistent with our findings. However, to our knowledge, no studies have specifically explored how weight status moderates associations between dietary intake and hypertension in adolescents. In this research, we noticed that even though meat, vegetable, and milk consumption were similar among adolescents of different weight status, the relationship between these food groups and high BP was dependent on the overweight status of the participants. In particular, vegetable and milk intake were related to decreased odds of hypertension while meat intake was related to increased odds of hypertension among overweight participants and these relationships were not observed in normal weight participants. With overweight adolescents already at increased odds of hypertension, these results indicated that higher vegetable and milk intake with lower meat intake could further decrease their likelihood of developing hypertension.

The major strength of this study is that we utilised both dietary habits questionnaire data and multiple-pass 24-h diet recalls, to estimate usual dietary intake. Moreover, all interviewers were trained to follow a strict protocol to minimise measurement error, thus improving accuracy. Furthermore, with accurate measurements of BMI, we were able to assess the moderating effects of weight status. This enabled us to disentangle the relationship between the diet and body adiposity to determine if diet can modify the risk of hypertension over and above body weight, though it should be noted that our sample consisted of multiple ethnicities where the appropriateness of overweight definitions may vary. There were several limitations in our study. Firstly, our data collection was interrupted due to COVID-19 lockdown and therefore a smaller sample size was recruited than hoped for. However, with 100 participants, regression models with several predictor variables can still be reliably estimated [40], although the exploratory moderating analyses should be interpreted with caution. Secondly, sampling bias may be present as we did not utilise a random sample. Thus, the sample does not represent the general NZ male adolescent population. Thirdly, we could not adjust for well-established confounding factors for high BP such as salt intake and physical activity levels [9] because we did not undertake 24-h urinary sodium tests and our measures of physical activity (through accelerometry) were limited to only a few participants due to COVID-19 restrictions. Lastly, BP was only measured in a single day as opposed to the standardised multiple days [41].

## 5. Conclusions

Our findings indicated that fruit, vegetable, and milk intake were associated with lower BP in a sample of male adolescents. In overweight adolescents, vegetable and milk intake appeared protective against hypertension, but meat intake was related to increased odds of hypertension. These were not observed in normal weight participants, demonstrating that certain dietary factors may have more prominent effects on BP among overweight individuals. Future research should aim to explore and validate how these dietary factors interact; through large scale longitudinal studies and trials while considering potential confounders such as salt intake and physical activity levels.

## Figures and Tables

**Table 1:** Demographic characteristics, weight status, and blood pressure (*n* = 108).

Variable	N (%) ^a^	Range
Age, mean (SD) years	16.6 (0.7)	15.0 to 17.9
Deprivation ^b^, mean (SD)	4.9 (2.6)	1 to 10
Low	34 (32.7)	
Medium	47 (45.2)	
High	23 (22.1)	
Ethnicity		104 participants responded to this question
Māori	10 (9.6)	
Pacific	3 (2.9)	
Asian	35 (33.7)	
NZEO ^c^	56 (53.9)	
BMI *z*-score ^d^, mean (SD)	0.45 (1.10)	−1.88 to 3.42
Weight status ^d^		
Healthy weight	72 (66.7)	
Overweight	30 (27.8)	
Obese	6 (5.6)	
Systolic blood pressure, mean (SD) mmHg	124 (12)	88 to 154
Diastolic blood pressure, mean (SD) mmHg	65 (9)	45 to 89
Elevated blood pressure ^e^	63 (58.3)	
Hypertensive ^e^	40 (37.0)	

^a^ Unless otherwise specified; ^b^ Deprivation assessed using area-level deprivation by applying home address to deciles of the New Zealand Deprivation Index 2018 (NZDep2018). Low deprivation: deciles 1 to 3; medium deprivation: deciles 4 to 7; high deprivation: deciles 8 to 10. Four participants were missing deprivation data; ^c^ NZEO—New Zealand European and others. Four participants were missing ethnicity data; ^d^ BMI *z*-scores determined using WHO growth charts. Overweight classified as BMI *z*-score >1 and ≤2; Obese classified as BMI *z*-score >2; ^e^ Elevated blood pressure as defined by the American Academy of Pediatrics: ≥120 mmHg systolic and <80 mmHg diastolic; Hypertensive as defined as: ≥130 mmHg systolic or ≥80 mmHg diastolic.

**Table 2:** Dietary habits and intake related to systolic and diastolic blood pressure.

	*n*	Mean (SD)	Mean Difference (95% CI) ^a^, mmHg	*p*-Value	Adjusted Mean Difference (95% CI) ^a^, mmHg	*p*-Value
Systolic blood pressure						
Dietary habits ^b^, serves per day						
Fruit	97	1.4 (1.1)	−2.2 (−4.3, −0.1)	0.040	−2.4 (−4.0, −0.7)	0.005
Vegetables	97	1.6 (1.2)	0.1 (−1.9, 2.1)	0.935	−0.7 (−2.4, 0.9)	0.370
Milk	97	1.3 (1.1)	−0.6 (−2.8, 1.6)	0.573	−0.7 (−2.5, 1.0)	0.410
Meat	96	2.2 (1.2)	−0.4 (−2.4, 1.6)	0.702	−1.3 (−2.9, 0.3)	0.110
Plant protein ^c^	96	1.5 (1.3)	0.8 (−0.9, 2.6)	0.352	0.7 (−0.7, 2.1)	0.352
Sweet drinks ^d^	97	1.1 (1.6)	0.4 (−1.0, 1.9)	0.574	0.4 (−0.9, 1.7)	0.519
Snacks ^e^	97	2.0 (1.8)	−1.3 (−2.5, −0.04)	0.043	−0.3 (−1.4, 0.7)	0.532
Daily dietary intake ^f^						
Energy, MJ	88	10.7 (3.9)	−0.2 (−0.9, 0.5)	0.627	−0.1 (−0.7, 0.5)	0.693
Protein, % energy	88	18.3 (3.2)	0.1 (−0.7, 1.0)	0.736	−0.2 (−0.9, 0.5)	0.517
Fat, % energy	88	37.6 (6.0)	0.0 (−0.4, 0.4)	0.998	−0.1 (−0.5, 0.3)	0.562
Carbohydrate, % energy	88	45.6 (6.8)	−0.2 (−0.6, 0.2)	0.393	0.1 (−0.3, 0.4)	0.685
Fibre, g	88	25.8 (11.2)	−0.1 (−0.3, 0.2)	0.506	−0.2 (−0.5, 0.2)	0.308
Diastolic blood pressure						
Dietary habits ^b^, serves per day						
Fruit	97	1.4 (1.1)	−2.5 (−4.0, −0.9)	0.002	−2.5 (−3.9, −1.1)	0.001
Vegetables	97	1.6 (1.2)	−1.1 (−2.6, 0.5)	0.174	−1.4 (−2.8, −0.02)	0.047
Milk	97	1.3 (1.1)	−2.2 (−3.9, −0.6)	0.007	−2.2 (−3.6, −0.8)	0.003
Meat	96	2.2 (1.2)	−0.4 (−1.9, 1.2)	0.629	−1.2 (−2.6, 0.2)	0.083
Plant protein ^c^	96	1.5 (1.3)	1.4 (0.01, 2.7)	0.048	1.1 (−0.04, 2.3)	0.058
Sweet drinks ^d^	97	1.1 (1.6)	0.3 (−0.8, 1.5)	0.567	0.2 (−0.9, 1.3)	0.701
Snacks ^e^	97	2.0 (1.8)	−0.9 (−1.9, 0.1)	0.066	−0.3 (−1.2, 0.6)	0.549
Daily dietary intake ^f^						
Energy, MJ	88	10.7 (3.9)	−0.4 (−1.0, 0.1)	0.102	−0.5 (−1.0, −0.04)	0.034
Protein, % energy	88	18.3 (3.2)	0.4 (−0.2, 1.0)	0.210	0.2 (−0.3, 0.8)	0.378
Fat, % energy	88	37.6 (6.0)	0.1 (−0.2, 0.5)	0.412	0.1 (−0.2, 0.4)	0.439
Carbohydrate, % energy	88	45.6 (6.8)	−0.3 (−0.6, 0.02)	0.065	−0.2 (−0.5, 0.1)	0.144
Fibre, g	88	25.8 (11.2)	−0.2 (−0.4, −0.01)	0.037	−0.2 (−0.5, 0.0)	0.074

^a^ Mean differences (95% CI) estimated using a mixed effects regression model, with school as a random effect. Adjusted estimates were adjusted for age, ethnicity, and BMI *z*-score. Fibre intake was also adjusted for energy intake; ^b^ Dietary habits assessed using the dietary habits questionnaire and measures frequency of intake; ^c^ Plant protein includes legumes, chickpeas, kidney beans, baked beans, tofu, tempeh, tofu products, vegetarian ingredients (such as Quorn, nut meat, vegetarian mince), vegetarian sausages, nuggets, patties, vegetarian “meat alternatives” (such as chicken-free chicken, vegetarian chicken schnitzel, meat-free bacon rashers); ^d^ Sweet drinks includes “diet” drinks but excludes alcoholic beverages (sensitivity analysis looking at diet sweet drinks separately saw a small change but did not influence estimates markedly: adjusted mean difference (95% CI): systolic 1.2 (−3.4, 5.8) mmHg, *p* = 0.611; diastolic 0.5 (−3.5, 4.5) mmHg, *p* = 0.802); ^e^ Snacks includes lollies, sweets, chocolate, confectionary, biscuits, cakes, muffins, sweet pastries, muesli or nut bars, and savoury snacks such as crisps and crackers; ^f^ MJ: Megajoules; Dietary intake assessed using 24-h recalls, adjusted for usual intake. Three participants are missing ethnicity data and are not included in the adjusted estimates.

**Table 3:** Demographic and dietary habits and the odds of hypertension (*n* = 97).

	Not Hypertensive (*n* = 62)	Hypertensive (*n* = 35)	Odds Ratio (95% CI) ^a^	*p*-Value	Adjusted Odds Ratio (95% CI) ^a^	*p*-Value
Age, years	16.5 (0.7)	16.9 (0.6)	2.62 (1.72, 3.99)	<0.001	3.16 (2.39, 4.19)	<0.001
Age group, *n* (%)						
15 to <17 years	48 (72.7)	18 (27.3)	Reference		Reference	
17 to <18 years	14 (45.2)	17 (54.8)	3.24 (1.62, 6.47)	0.001	3.54 (1.61, 7.84)	0.002
Deprivation, *n* (%)						
Low	24 (72.7)	9 (27.3)	Reference		Reference	
Medium	27 (62.8)	16 (37.2)	1.58 (0.38, 6.54)	0.528	1.28 (0.41, 4.04)	0.670
High	11 (52.4)	10 (47.6)	2.42 (0.54, 10.83)	0.246	0.94 (0.32, 2.74)	0.914
Ethnicity, *n* (%)						
NZEO	36 (64.3)	20 (35.7)	Reference		Reference	
Māori	6 (60.0)	4 (40.0)	1.20 (0.28, 5.12)	0.806	1.21 (0.45, 3.27)	0.701
Asian	20 (64.5)	11 (35.5)	0.99 (0.46, 2.15)	0.980	2.05 (0.98, 4.29)	0.058
BMI *z*-score, mean (SD)	0.04 (0.99)	1.15 (1.03)	3.01 (1.03, 8.76)	0.044	3.24 (1.08, 9.69)	0.036
Weight status, *n* (%)						
Not overweight	51 (79.7)	13 (20.3)	Reference		Reference	
Overweight	11 (33.3)	22 (66.7)	7.85 (2.54, 24.16)	<0.001	9.42 (2.89, 30.69)	<0.001
Dietary habits ^b^, median (25th, 75th percentiles) serves per day						
Fruit	1 (0.4, 2)	1 (0.4, 2)	0.84 (0.54, 1.28)	0.410	0.69 (0.40, 1.19)	0.183
Vegetables	1 (0.8, 2)	1 (0.8, 2)	1.03 (0.76, 1.39)	0.858	0.84 (0.59, 1.18)	0.316
Milk	1 (0.4, 2.5)	1 (0.4, 2.5)	1.14 (0.78, 1.67)	0.497	1.12 (0.67, 1.87)	0.663
Meat	1.8 (1.4, 2.7)	2.0 (1.5, 2.6)	1.11 (0.92, 1.34)	0.256	0.96 (0.64, 1.46)	0.862
Plant protein ^c^	0.8 (0.4, 2.5)	0.7 (0.2, 3.5)	1.05 (0.88, 1.25)	0.583	1.03 (0.66, 1.60)	0.901
Sweet drinks ^d^	0.6 (0.1, 1.2)	0.8 (0.1, 1.3)	1.13 (1.03, 1.24)	0.008	1.16 (0.998, 1.34)	0.053
Snacks ^e^	1.6 (0.8, 2.6)	1.3 (0.6, 2.1)	0.94 (0.81, 1.09)	0.399	1.09 (0.83, 1.42)	0.548
Daily dietary intake ^f^	*N* = 55	*N* = 33				
Energy, MJ	11.0 (4.3)	10.3 (3.2)	0.95 (0.89, 1.01)	0.078	0.88 (0.74, 1.05)	0.164
Protein, % energy	18.3 (3.3)	18.2 (3.0)	0.99 (0.85, 1.15)	0.893	0.94 (0.79, 1.12)	0.491
Fat, % energy	32.3 (6.2)	38.0 (5.8)	1.02 (0.96, 1.09)	0.534	1.04 (0.96, 1.12)	0.340
Carbohydrate, % energy	46.0 (6.1)	44.9 (8.0)	0.98 (0.94, 1.01)	0.199	1.00 (0.93, 1.06)	0.884
Fibre, g	27.1 (11.6)	23.6 (10.3)	0.97 (0.94, 0.997)	0.033	0.93 (0.85, 1.02)	0.117

^a^ Odds ratios (95% CI) estimated using a logistics regression model, accounting for school with a sandwich estimator. Adjusted estimates were adjusted for age and BMI *z*-score. Fibre intake was also adjusted for energy intake; ^b^ Dietary habits assessed using the dietary habits questionnaire and measures frequency of intake; ^c^ Plant protein includes legumes, chickpeas, kidney beans, baked beans, tofu, tempeh, tofu products, vegetarian ingredients (such as Quorn, nut meat, vegetarian mince), vegetarian sausages, nuggets, patties, vegetarian “meat alternatives” (such as chicken-free chicken, vegetarian chicken schnitzel, meat-free bacon rashers); ^d^ Sweet drinks includes “diet” drinks but excludes alcoholic beverages (sensitivity analysis showed a significantly lower odds for each non-diet drink extra per day: adjusted odds ratio (95% CI); 0.85 (0.72, 1.00), *p* = 0.049); ^e^ Snacks includes lollies, sweets, chocolate, confectionary, biscuits, cakes, muffins, sweet pastries, muesli or nut bars, and savoury snacks such as crisps and crackers; ^f^ Dietary intake assessed using 24-h recalls, adjusted for usual intake.

## Data Availability

The data presented in this study are available on request from the corresponding author.

## Appendix A

**Table A1:** Differences in demographics and habits by age group and weight status.

	Age Group			Weight Status		
	15 to <17 Years (*n* = 66)	17 to <18 Years (*n* = 31)	*p*-Value ^a^	Not Overweight (*n* = 64)	Overweight (*n* = 33)	*p*-Value ^a^
Age, years	16.2 (0.5)	17.5 (0.3)	-	16.6 (0.8)	16.7 (0.6)	0.541
Age group, *n* (%)			-			0.502
15 to <17 years	66 (100)	0		45 (68.2)	21 (31.8)	
17 to <18 years	0	31 (100)		19 (61.3)	12 (38.7)	
Deprivation, *n* (%)			0.961			0.003
Low	22 (66.7)	11 (33.3)		25 (75.8)	8 (24.2)	
Medium	30 (69.8))	13 (30.2)		32 (74.4)	11 (24.6)	
High	14 (66.7)	7 (33.3)		7 (33.3)	14 (66.7)	
Ethnicity, *n* (%)			0.406			0.350
NZEO	36 (64.3)	20 (35.7)		36 (64.3)	20 (35.7)	
Māori	6 (60.0)	4 (40.0)		5 (50.0)	5 (50.0)	
Asian	24 (77.4)	7 (22.6)		23 (74.2)	8 (25.8)	
BMI *z*-score, mean (SD)	0.36 (1.10)	0.60 (1.20)	0.323	-0.21 (0.70)	1.69 (0.64)	-
Weight status, *n* (%)			0.502			-
Not overweight	45 (70.3)	19 (29.7)		64 (100)	0	
Overweight	21 (63.6)	12 (36.4)		0	33 (100)	
Dietary habits ^b^, mean (SD) serves per day						
Fruit	1.4 (1.1)	1.5 (1.1)	0.816	1.3 (1.0)	1.6 (1.2)	0.371
Vegetables	1.5 (1.1)	1.8 (1.2)	0.173	1.5 (1.1)	1.8 (1.4)	0.174
Milk	1.2 (1.0)	1.5 (1.3)	0.233	1.2 (1.1)	1.5 (1.1)	0.121
Meat	2.0 (1.2)	2.5 (1.2)	0.052	2.1 (1.3)	2.3 (1.0)	0.461
Plant protein ^c^	1.4 (1.3)	1.5 (1.4)	0.849	1.5 (1.2)	1.4 (1.5)	0.712
Sweet drinks ^d^	1.0 (1.4)	1.2 (2.0)	0.648	1.0 (1.3)	1.2 (2.1)	0.615
Snacks ^e^	2.1 (2.0)	1.8 (1.4)	0.400	2.0 (1.7)	1.9 (2.1)	0.656
Daily dietary intake ^f^	*N* = 56	*N* = 32		*N* = 58	*N* = 30	
Energy, MJ	10.4 (4.0)	11.3 (3.7)	0.274	10.1 (2.7)	11.8 (5.5)	0.055
Protein, % energy	18.0 (3.4)	18.7 (2.7)	0.311	18.3 (3.1)	18.3 (3.3)	0.976
Fat, % energy	37.9 (6.6)	37.0 (4.9)	0.505	37.3 (6.3)	38.0 (5.6)	0.630
Carbohydrate, % energy	46.0 (7.2)	45.0 (6.1)	0.519	46.2 (6.5)	44.5 (7.4)	0.272
Fibre, g	24.3 (9.9)	28.4 (12.9)	0.105	25.2 (9.8)	27.0 (13.7)	0.476

^a^ *p*-value calculated with a t-test for continuous variables or a Fisher’s exact test for categorical variables; ^b^ Dietary habits assessed using the dietary habits questionnaire and measures *frequency* of intake; ^c^ Plant protein includes legumes, chickpeas, kidney beans, baked beans, tofu, tempeh, tofu products, vegetarian ingredients (such as Quorn, nut meat, vegetarian mince), vegetarian sausages, nuggets, patties, vegetarian “meat alternatives” (such as chicken-free chicken, vegetarian chicken schnitzel, meat-free bacon rashers); ^d^ Sweet drinks includes “diet” drinks but excludes alcoholic beverages (sensitivity analysis showed a significantly lower odds for each non-diet drink extra per day: adjusted odds ratio (95% CI); 0.85 (0.72, 1.00), *p* = 0.049); ^e^ Snacks includes lollies, sweets, chocolate, confectionary, biscuits, cakes, muffins, sweet pastries, muesli or nut bars, and savoury snacks such as crisps and crackers; ^f^ MJ: Megajoules; Dietary intake assessed using 24-h recalls, adjusted for usual intake.
